# Intracellular metabolite profiling of *Saccharomyces cerevisiae* evolved under furfural

**DOI:** 10.1111/1751-7915.12465

**Published:** 2016-12-08

**Authors:** Young Hoon Jung, Sooah Kim, Jungwoo Yang, Jin‐Ho Seo, Kyoung Heon Kim

**Affiliations:** ^1^ School of Food Science and BiotechnologyKyungpook National UniversityDaegu41566South Korea; ^2^ Department of BiotechnologyGraduate SchoolKorea UniversitySeoul02841South Korea; ^3^ Department of Agricultural Biotechnology and Center for Food and BioconvergenceSeoul National UniversitySeoul08826South Korea

## Abstract

Furfural, one of the most common inhibitors in pre‐treatment hydrolysates, reduces the cell growth and ethanol production of yeast. Evolutionary engineering has been used as a selection scheme to obtain yeast strains that exhibit furfural tolerance. However, the response of *Saccharomyces cerevisiae* to furfural at the metabolite level during evolution remains unknown. In this study, evolutionary engineering and metabolomic analyses were applied to determine the effects of furfural on yeasts and their metabolic response to continuous exposure to furfural. After 50 serial transfers of cultures in the presence of furfural, the evolved strains acquired the ability to stably manage its physiological status under the furfural stress. A total of 98 metabolites were identified, and their abundance profiles implied that yeast metabolism was globally regulated. Under the furfural stress, stress‐protective molecules and cofactor‐related mechanisms were mainly induced in the parental strain. However, during evolution under the furfural stress, *S. cerevisiae* underwent global metabolic allocations to quickly overcome the stress, particularly by maintaining higher levels of metabolites related to energy generation, cofactor regeneration and recovery from cellular damage. Mapping the mechanisms of furfural tolerance conferred by evolutionary engineering in the present study will be led to rational design of metabolically engineered yeasts.

## Introduction

Lignocellulose is the most abundant and promising resource for producing fuels and bio‐based chemicals. To efficiently produce fermentable sugars from lignocellulose, lignocellulose must be pre‐treated because of its high recalcitrance. However, the generation of various degradation by‐products, including 2‐furaldehyde (furfural), 5‐hydroxymethyl‐2‐furaldehyde, organic acids and phenolics, which negatively affect microbial metabolism during fermentation, is unavoidable (Liu, 2006; Almeida *et al*., 2009; Jung *et al*., 2014). It is because physicochemical pretreatments are performed at extreme conditions such as high temperatures and/or extreme pH values. Furthermore, furfural, which is derived from pentose sugars, is known to be one of the most potent contributors to the toxicity of pretreatment hydrolysates for fermentative microorganisms (Heer and Sauer, 2008). Furfural significantly reduces cell proliferation and ethanol production either by inhibiting several enzymes that are essential to central metabolism, including dehydrogenases, or by damaging and blocking the synthesis of DNA, RNA, protein and cell wall (Zaldivar *et al*., 1999; Modig *et al*., 2002; Horváth *et al*., 2003; Almeida *et al*., 2009; Liu, 2011; Ask *et al*., 2013; Wilson *et al*., 2013). Fortunately, unless the furfural level is lethal, *Saccharomyces cerevisiae* can metabolize it into less toxic compounds such as furfuryl alcohol and furoic acid by consuming NAD(P)H at the beginning of fermentation (Liu *et al*., 2004; 2005).

Various strategies to ameliorate furfural toxicity, including the physical and chemical detoxification of hydrolysates prior to fermentation, have been investigated (Palmqvist and Hahn‐Hägerdal, 2000; Jung and Kim, 2014). However, because of the high cost of the detoxification processes, strategies to enhance the inherent resistance of microbes to furfural have been receiving much attention. Many efforts have been made to develop furfural‐resistant fermentative strains. For example, the pentose phosphate pathway, γ‐aminobutyric acid (GABA) shunt, cofactor interconversion, high osmolality glycerol signalling and DNA binding processes seem to be associated with growth improvement under furfural stress (Modig *et al*., 2002; Gorsich *et al*., 2006; Kim *et al*., 2012; Wang *et al*., 2013a; Glebes *et al*., 2015). Several genes under furfural stress, which are involved in stress tolerance (e.g. dehydrogenases), cofactor balance (e.g. oxidoreductases and transhydrogenase) and other functions (e.g. sulfur assimilation and glucose phosphorylation), have been identified (Nilsson *et al*., 2005; Liu, 2006; 2011; Heer *et al*., 2009; Miller *et al*., 2009; Yang *et al*., 2012; Wilson *et al*., 2013). The production of reactive oxygen species (ROS) and changes in energy status also influence cellular physiology of *S. cerevisiae* (Allen *et al*., 2010; Ask *et al*., 2013).

In recent years, evolutionary engineering of microbes, which relies on selective pressure towards an appropriate phenotype, has also been investigated. For example, through evolution in the presence of furfural, *S. cerevisiae* showed improvement in tolerance to furfural toxicity and in the ability to convert furfural to less toxic materials (Heer *et al*., 2009; Liu *et al*., 2009). The regulation of central carbon metabolism, redox balance, membrane fatty acids and amino acids in the presence of furfural has been investigated at the proteomic, lipidomic and metabolomic levels to determine the effects of these components on furfural tolerance in yeast (Lin *et al*., 2009; Xia and Yuan, 2009; Ding *et al*., 2011; Wang *et al*., 2013b). In particular, through metabolic profiling of yeast adapted in lignocellulosic hydrolysates containing multiple inhibitors including furfural, alanine, GABA and glycerol have been suggested as the key metabolites (Wang *et al*., 2013b). However, as microbial metabolism is tightly and globally regulated by a large number of intracellular metabolites following mass and energy conservation laws (Patil *et al*., 2005), metabolic approaches for an individual compound need to be more thoroughly investigated.

Presently, there are insufficient evolutionary engineering studies regarding the strategies adopted by yeast for coping with furfural at the metabolite level. In this study, responses of both the parental strain and the evolved yeast for furfural were studied by analysing all the intracellular metabolites. First, an evolutionary engineering strategy in the presence of furfural stress was applied to *S. cerevisiae* to improve its tolerance for many generations. Second, the physiological basis of furfural resistance was explored by a match‐up of the fermentation profiles of both the parental and the evolved strains. Finally, global profiles of the metabolites expressed in the parental and the evolved yeast were obtained using gas chromatography/time‐of‐flight mass spectrometry (GC/TOF MS) and compared. This study explored the metabolic perturbation patterns of *S. cerevisiae* both when the yeast encountered furfural by chance and when it was intentionally adapted to furfural.

## Results and discussion

### Evaluation of *S. cerevisiae* D_5_A under furfural

**Figure 1:**
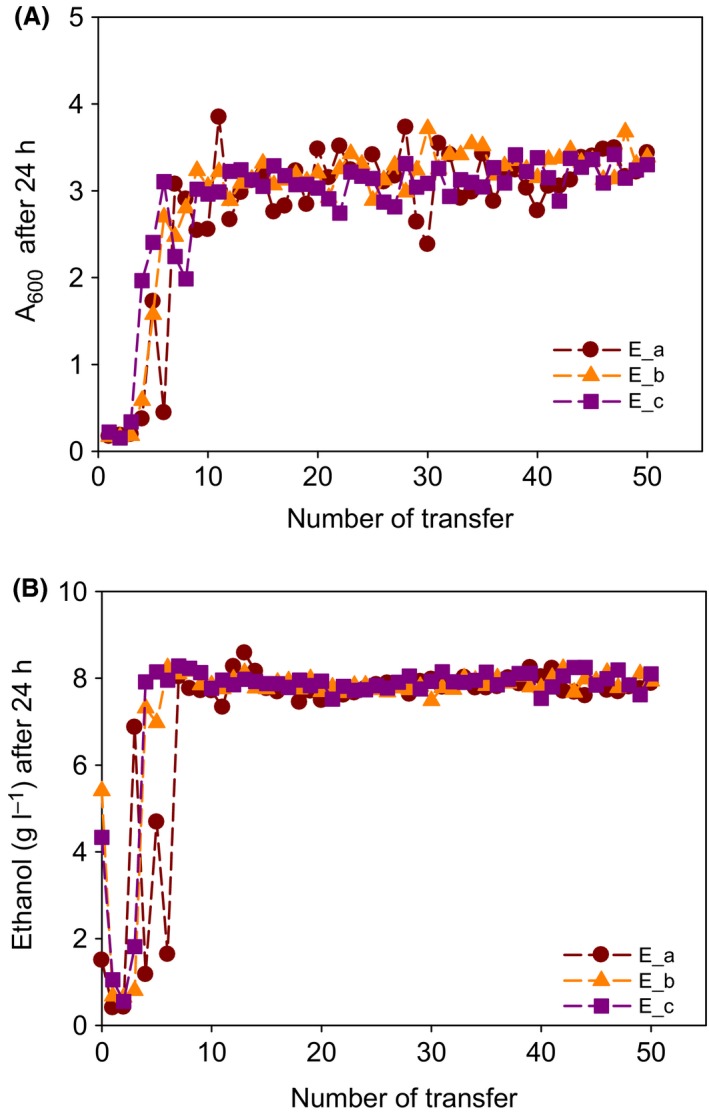
Profiles of (A) cell growth (A_600_, absorbance at 600 nm) and (B) ethanol production. For evolution, three seed cultures of *Saccharomyces cerevisiae* were independently grown in YPD medium containing 20 mM furfural at 30°C and 200 r.p.m. *Saccharomyces cerevisiae* D_5_A was transferred after 24 h of cultivation under furfural during 50 transfers (~332 generations).

To obtain evolved yeast strains which are tolerant to furfural, three different seed cultures of *S. cerevisiae* D_5_A (i.e. E_a, E_b and E_c) were cultivated independently in different tubes and transferred 50 times to fresh media containing 20 mM furfural. Cell concentrations and ethanol titres of the culture at each transfer were measured after 24 h of cultivation to monitor the evolutionary progress (Fig. 1). After approximately 5–10 transfers, cell growth and ethanol production in the presence of 20 mM furfural significantly increased. These rapid adaptation patterns have also been observed by other groups. For example, the long lag phases induced due to the presence of furfural were effectively shortened by evolution in two transfers under about 13.5 mM furfural (Wang *et al*., 2013b) or by 20 transfers under 17 mM furfural (Heer and Sauer, 2008). To verify whether the phenotype of furfural tolerance in the evolved strains was maintained in the absence of furfural, the evolved strains (E_a, E_b and E_c) were transferred for approximately 33 generations in YPD medium without furfural and was then recultivated with furfural (Fig. S1). When the evolved strains that were maintained in the absence of furfural were returned to furfural‐containing media, its growth was still much higher than that of the parental strain. These results indicated a fixation of the phenotype in the evolved strains.

Next, we compared the cell growth behaviour of the evolved strains with that of the parental strain. In the presence of furfural (0–40 mM), the lag phase of *S. cerevisiae* D_5_A increased from 2.5 to 45 h for the parental strain and from 3 to 28 h for the evolved strains as the furfural level increased from 0 to 30 mM (Fig. S2). This increase was probably due to furfural‐induced inhibition of key enzymes in the glycolytic pathway (Palmqvist *et al*., 1999; Horváth *et al*., 2003; Wang *et al*., 2013b). In addition, under 20 mM furfural, compared with the parental strain, the evolved strains grew extremely fast (Fig. S3). Accordingly, around 20 g l^−1^ glucose and furfural were consumed within 18 h of fermentation by the evolved strains, and ethanol was produced at a rate of 0.3 g ethanol g^−1^ dry cell weight per hour to a maximum yield of 0.9 g g^−1^ after 18 h of fermentation (Table 1).

**Table 1:** Comparison of physiological values of the parental and evolved strains grown in YPD medium with or without 20 mM furfural. For the evolved strains, the mean values of E_a, E_b and E_c were used

	Parental strain		Evolved strains	
Furfural concentration (mM)	0	20	0	20
Max. specific growth rate (h^−1^)	0.37	0.19	0.23	0.24
Cell dry weight at 48 h (g l^−1^)	3.2	2.0	2.9	2.7
Glucose depletion time (h)	9	30	15	18
Glucose consumption rate (g g^−1^ DCW h^−1^)	1.9	0.8	1.5	0.6
Furfural consumption rate (g g^−1^ DCW h^−1^)	NA	0.4	NA	0.3
Ethanol production rate (g g^−1^ DCW h^−1^)	0.9	0.3	0.7	0.3

### PCA of intracellular metabolites of the evolved strains versus the parental strains

**Figure 2:**
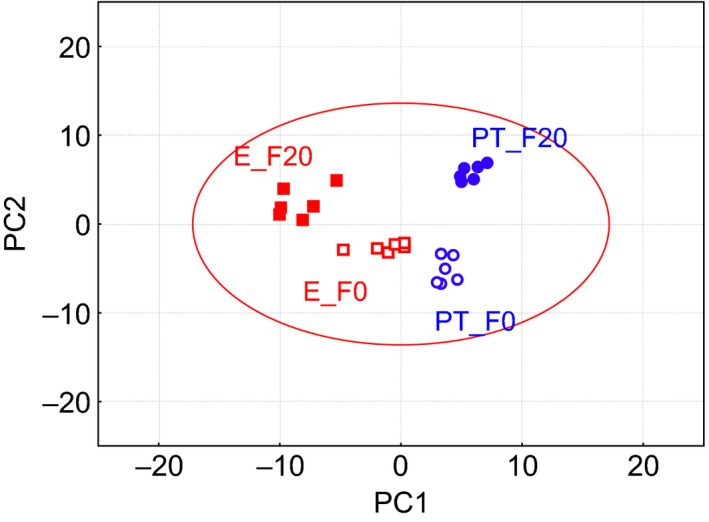
Principal component analysis of identified metabolites in the parental and evolved strains. Both strains were grown in YPD medium with or without 20 mM furfural. PT_F0: the parental strain without furfural; PT_F20: the parental strain with 20 mM furfural; E_F0: the evolved strains without furfural; E_F20: the evolved strains with furfural. For the evolved strains, the mean values of E_a, E_b and E_c were used.

Identifying the cellular metabolic reactions to environmental changes at the metabolite level is of great interest. In this study, six replicates of the parental *S. cerevisiae* strains and duplicates of the three evolved *S. cerevisiae* strains, which were grown with or without furfural, were collected at the early exponential phase for metabolite analysis. A total of 98 meaningful metabolites from different classes, including amines and phosphates, amino acids, fatty acids and phenolics, organic acids, and sugars and sugar alcohols, were identified (Table S1). To provide comparative information regarding the metabolomic differences among the four groups, principal component analysis (PCA) was performed. The differences among the four groups were well explained by the PCA model, which showed an explained variation value (*R* ^2^ *X*) of 0.94 and a predictive capability (*Q* ^2^) of 0.93. Although the first principal component (PC1) and the second principal component (PC2) showed less discrimination (*R* ^2^ *X* = 0.52 and *Q* ^2^ = 0.46), PC1 and PC2 appeared to be the major variation factors induced by evolution and by furfural respectively (Fig. 2). Accordingly, on the basis of the differential distribution reflecting the importance of the original variables, it could be expected that metabolites with high loading in PC1 were related either to the glutathione and the thioredoxin reduction system (e.g. homoserine, cysteine, glutamate and 5′‐deoxy‐5′‐methylthioadenosine) for relieving ROS accumulation or to the sugar metabolisms (e.g. glucose, galactose, fructose and mannose) to maintain energy balance. Conversely, cofactor‐related metabolites (e.g. phenylacetate, salicylaldehyde and 3‐hydroxypropionate) and amino acids (e.g. lysine, *N*‐methylalanine, proline, glycine and threonine) were found to be relatively predominant in PC2 (Table S2). Several fatty acids, including palmitoleic acid, pentadecanoic acid and palmitic acid, did not contribute to the clear separation among the four groups. These results suggest that the intracellular fatty acid metabolism was not significantly affected by either the furfural stress or the evolutionary engineering.

### Metabolic traits of the evolved strains under furfural stress

Biological interpretation of the identified metabolites is crucial for better understanding of the functional metabolism as a means of coping with furfural stress. In this study, after categorical annotation of the identified metabolites into suitable groups (Table S1), the value of each metabolite was normalized by the sum of peak intensities of all the detected intracellular metabolites, which were analysed by GC/TOF MS, from each culture. Next, the average values of normalized data from both the parental and evolved strains grown under furfural stress were compared with the data from those grown without furfural to analyse the effect of furfural. Significant differences in the set of normalized data were evaluated (*p *<* *0.05), and the variables without significant differences were considered to show similar expression levels, such as galactose in the parental strain and sucrose in the evolved strains, regardless of abundance changes. Relative comparison of the obtained fold changes was introduced to explain the metabolic fates caused by the evolutionary process.

The metabolic fates of parental and evolved *S. cerevisiae* grown with or without furfural stress were thoroughly investigated by selection procedures using stringency criteria (fold changes and *p* values). In this study, overall, the principal regulation mechanisms for coping with furfural toxicity differed markedly between the parental strain and the evolved strains. The parental strain tried to minimize primary metabolism and maximized the production of stress‐related metabolites in response to furfural; the evolved strains, which was already habituated to the reduced environment, seemed to attempt to restore the anabolism suppressed by furfural. Specifically, we investigated carbohydrate metabolism, amino acid synthesis and cofactor‐related pathways.

**Figure 3:**
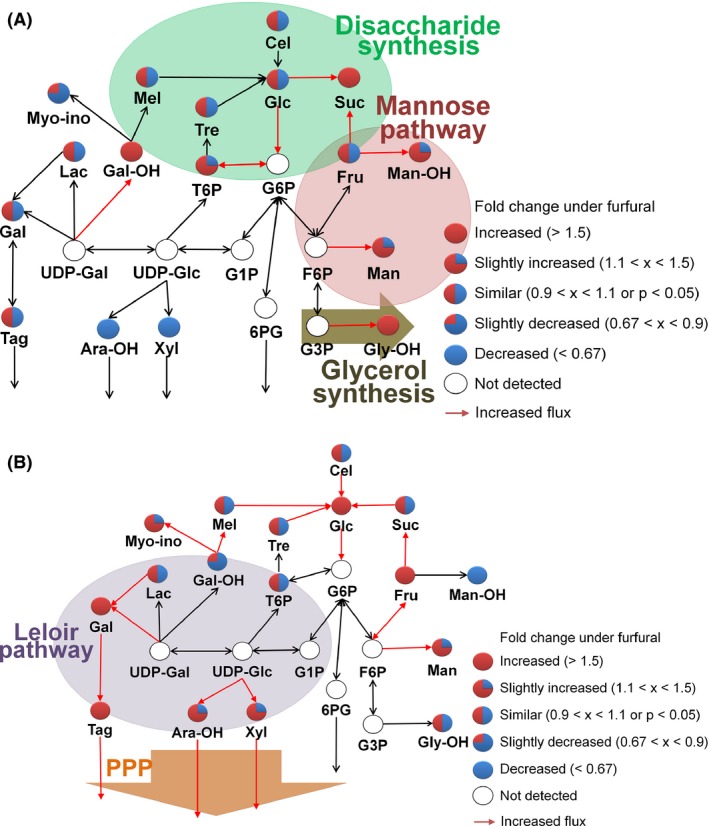
The carbohydrate metabolic pathways in the (A) parental and (B) evolved strains. For the evolved strains, the mean values of E_a, E_b and E_c were used. The fold changes indicate the fold increases of metabolite abundances under the furfural stress in comparison with the metabolite abundances without furfural stress in the parental strain or the evolved strains. 6PG, 6‐phosphogluconate; Ara‐OH; arabinol; Cel, cellulose; Fru, fructose; F6P, fructose‐6‐phosphate; Gal, galactose; Gal‐OH, galactinol; Glc, glucose; G1P, glucose‐1‐phosphate; G3P, glyceraldehyde‐3‐phosphate; G6P, glucose‐6‐phosphate; Gly‐OH, glycerol; Lac, lactose; Man, mannose; Man‐OH, mannitol; Mel, melibiose; Myo‐ino, myo‐inositol; Suc, sucrose; Tag, tagatose; Tre, trehalose; T6P, trehalose‐6‐phosphate; UDP‐Gal, uridine diphosphate galactose; UDP‐Glc, uridine diphosphate glucose; Xyl, xylose.

The central carbon metabolic pathway appeared to differ between the parental and evolved *S. cerevisiae* (Fig. 3). In the parental strain, sucrose, trehalose‐6‐phosphate, mannose, glycerol and others were higher in the furfural stress than those without furfural stress. As the presence of stress represses the expression of several enzymes in glycolysis, including aldehyde dehydrogenase, alcohol dehydrogenase and pyruvate dehydrogenase, a problem occurs in the generation of energy and building blocks (Cadière *et al*., 2011). Thus, the higher abundance of glycerol may have acted as a protectant under the furfural stress (Wang *et al*., 2013b). In addition, under stressful environments, yeasts must reduce ATP demands to recover from substrate‐accelerated death caused by the imbalance between energy production and consumption. Thus, an ATP futile cycle through sugar phosphate and disaccharide synthesis would be induced to counteract the stress‐induced ATP imbalance (Francois and Parrou, 2001; Jansen *et al*., 2006). In this study, in the parental strain, the intracellular abundance of sucrose was higher as a safety valve under the furfural stress (Fig. 3A). On the other hand, in the evolved strains, monosaccharides such as glucose and fructose were higher, and various metabolites from the Leloir metabolism, including galactose, tagatose, xylose and arabitol, were significantly higher under the furfural stress (Fig. 3B). These results imply that in the evolved strains, the fluxes through the glycolytic and pentose phosphate pathways were recovered or intensified despite the furfural stress, probably to generate suitable amounts of energy, cofactors and other intermediate metabolites for the synthesis of aromatic amino acids and nucleotides.

**Figure 4:**
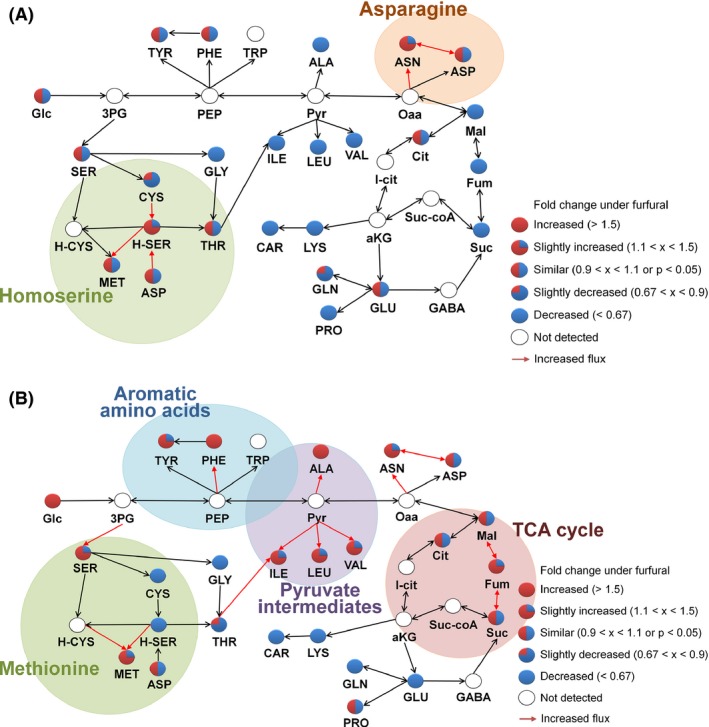
The amino acid synthesis pathways in the (A) parental and (B) evolved strains. For the evolved strains, the mean values of E_a, E_b and E_c were used. The fold changes indicate the fold increases of metabolite abundances under the furfural stress in comparison with the metabolite abundances without furfural stress in the parental strain or the evolved strains. 3PG, 3‐phosphoglycerate; aKG, α‐keto glutarate; ALA, alanine; ASN, asparagine; ASP, aspartate; CAR, carnitine; Cit, citrate; CYS, cysteine; Fum, fumarate; GABA, γ‐aminobutyric acid; Glc, glucose; GLN, glutamine; GLU, glutamate; GLY, glycine; H‐CYS, homocysteine; H‐SER, homoserine; I‐cit, Isocitrate; ILE, isoleucine; LEU, leucine; LYS, lysine; Mal, malate; MET, methionine; Oaa, oxaloacetate; PEP, phosphoenolpyruvate; PHE, phenylalanine; PRO, proline; Pyr, pyruvate; SER, serine; Suc, succinate; Suc‐coA, succinyl‐CoA; THR, threonine; TRP, tryptophan; TYR, tyrosine; VAL, valine.

With regard to the amino acid metabolism, in the parental strain, most of amino acids were lower in abundance under the furfural stress than those without furfural stress, possibly due to the shortage of energy resulting from the ATP futile cycle and due to the inhibition of primary metabolism under the furfural stress (Fig. 4A), as observed earlier in the carbohydrate metabolism (Fig. 3). In the evolved strains, the abundances of amino acids were significantly higher than those under the furfural stress (Fig. 4B), implying that glycolytic activity was restored over the course of evolution (Wang *et al*., 2013b). Accordingly, in the evolved strains under the furfural stress, the abundances of the TCA cycle intermediates were maintained at the levels similar to those in the evolved strains without furfural (Fig. 4). This phenomenon on the TCA cycle intermediates was unlike those in the parental strain. In the evolved strains, the abundances of branched chain amino acids such as isoleucine, valine and leucine were also higher under the furfural stress than those without furfural stress (Fig. 4B), possibly either due to the source of acetyl‐CoA or due to the substrates for alanine synthesis, which provide energy efficiency via the alanine‐GABA shunt and the alanine‐glucose cycle (Wang *et al*., 2013b). Meanwhile, in the evolved strains, the synthesis of glutamate and glutamine from ammonia in the central nitrogen metabolism was less active under the furfural stress (Fig. 4B). Probably, these reactions needed to decrease in order to save reduced cofactors as these reactions consume NAD(P)H.

**Figure 5:**
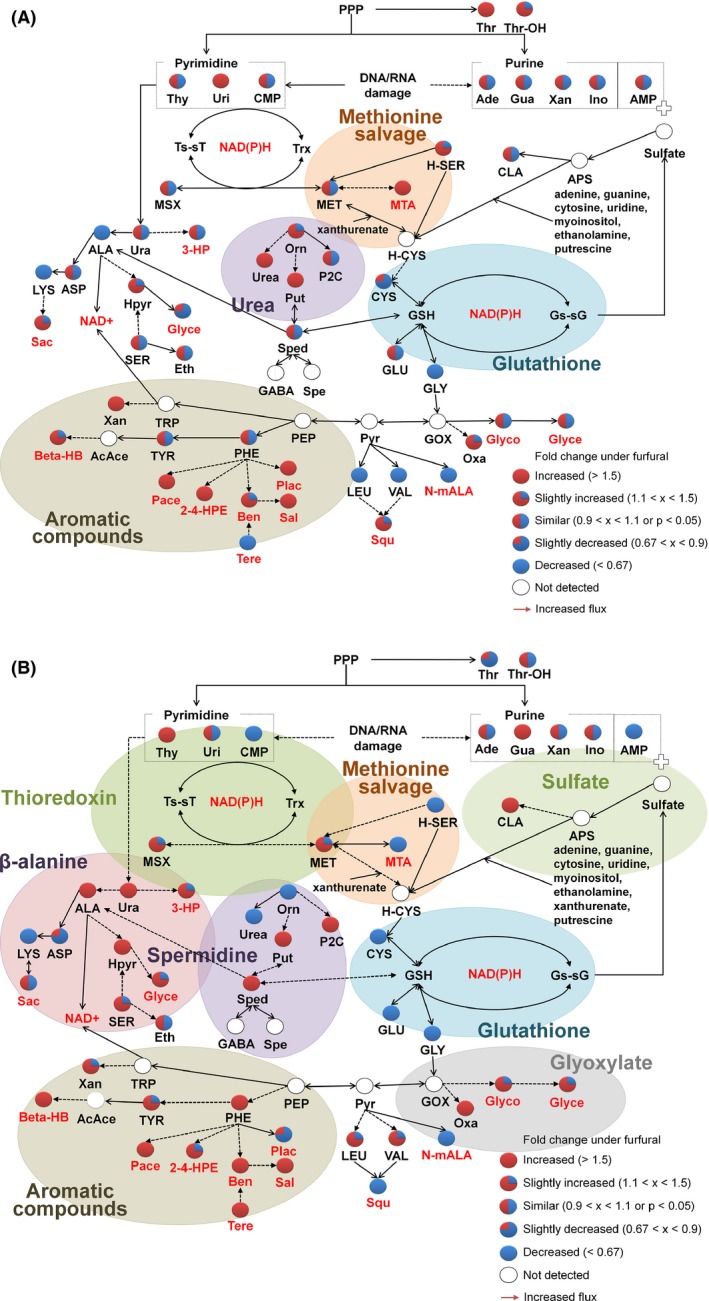
The NAD(P)H pool metabolism in the (A) parental and (B) evolved strains. For the evolved strains, the mean values of E_a, E_b and E_c were used. The fold changes indicate the fold increases of metabolite abundances under the furfural stress in comparison with the metabolite abundances without furfural stress in the parental strain or the evolved strains. Metabolites written in red color are directly related to cofactor regulation. 2‐4‐HPE, 2‐(4‐hydroxyphenyl)ethanol; 3‐HP, 3‐hydroxypropionate; AcAce, acetoacetate; Ade, adenosine; AMP, adenosine‐5′‐monophosphate; ALA, alanine; APS, adenosine‐5′‐phosphosulfate; ASP, aspartate; Ben, benzoate; Beta‐HB, β‐hydroxybutyrate; CLA, cyano‐l‐alanine; CMP, cytidine‐5′‐monophosphate; CYS, cysteine; Eth, ethanolamine; GABA, γ‐aminobutyric acid; GLU, glutamate; GLY, glycine; Glyce, glycerate; Glyco, glycolate; GOX, glyoxylate; GSH, glutathione; Gs‐sG, glutathione‐sulfur complex; Gua, guanine; H‐CYS, homocysteine; Hpyr, hydroxypyridine; H‐SER, homoserine; Ino, inosine; LEU, leucine; LYS, lysine; MET, methionine; MSX, methionine sulfoxide; MTA, 5′‐deoxy‐5′‐methyl thioadenosine; NAD+, nicotinamide adenine dinucleotide +; N‐mALA,* N*‐methylalanine; Orn, ornithine; Oxa, oxalate; P2C, pyrrole‐2‐carboxylate; Pace, phenylacetate; PEP, phosphoenolpyruvate; PHE, phenylalanine; Put, putrescine; Plac, phenyllactate; PPP, pentose phosphate pathway; Pyr, pyruvate; Sac, saccharopine; Sal, salicylaldehyde; SER, serine; Spe, spermine; Sped, spermidine; Squ, squalene; Tere, terephthalate; Thr, threose; Thr‐OH, threitol; Thy, thymine; TRP, tryptophan; Trx, thioredoxin; Ts‐sT, thioredoxin‐sulfur complex; TYR, tyrosine; Ura, uracil; Uri, uridine; VAL, valine; Xan, xanthine.

Finally, changes in the metabolite abundances in the redox system were thoroughly investigated, as the consumption of NAD(P)H is necessary to metabolize furfural into less toxic compounds such as furfuryl alcohol and furoic acid (Liu *et al*., 2004; 2005). Metabolism related to aromatic compounds originating from phenylalanine, including β‐hydroxybutyrate, phenylacetate, phenyllactate, hydroxyphenylethanol, benzoate and salicylaldehyde, was significantly intensified (Fig. 5), which is probably affecting the enhanced flux to the pentose phosphate pathway (Park *et al*., 2011). In the parental strain, due to lack of energy to cope with the furfural stress and damages to the protein, a stronger cofactor‐regenerating mechanism with urea excretion was indispensable under the furfural stress (Fig. 5A). However, in the evolved strains, increases in metabolite abundances were observed in various metabolites as a correspondence mechanism under the furfural stress. In the evolved strains, along with aromatic compounds, the abundances of metabolites in the β‐alanine cycle and several organic acids, including glycolate and glycerate, were higher possibly to secure NAD(P)H and/or acetyl‐CoA availability under the furfural stress (Fig. 5B). In addition, to recover from the DNA or RNA damage caused by furfural, the thioredoxin cycle was well managed in the evolved strains under the furfural stress (Fig. 5B), probably for the restoration of nucleotides (Carmel‐Harel and Storz, 2000; Shi *et al*., 2011). The abundances of both putrescine and spermidine, which can act as protectants from abiotic stress or as substrates related to the protein synthesis initiation factor (Shimogori *et al*., 1996; Gill and Tuteja, 2010), were higher under the furfural stress.

## Conclusions

Significant metabolic rearrangements in response to furfural stress were revealed in *S. cerevisiae* by a combination of evolutionary engineering and metabolomics. The formation of stress‐protective molecules, including glycerol and disaccharides, was important in maintaining the metabolic activity of the parental strain under furfural stress. Contrary to the *ad hoc* responses in the parental strain, the coping mechanisms in the evolved strains appeared to be strongly sessile throughout evolutionary engineering. After rapid adaptation and physiological stabilization, we explored metabolism, which was remarkably strengthened by the improvement of glycolytic activity, salvation of spermidine and methionine and restoration of NAD(P)H pools. In conclusion, when the yeast recognizes the presence of furfural stress, they may globally regulate their metabolic status in advance in response to the furfural stress. The comparisons of defence mechanisms against furfural in the parental and evolved *S. cerevisiae* in this study provide new insights into the systems biology of yeast physiology.

## Experimental procedures

### Strain, media, culture conditions and evolution experiments

The parental strain *S. cerevisiae* D_5_A (ATCC 200062) was used as a starting strain for the evolution experiments. Three evolved phenotypes were independently generated in separate culture tubes through serial transfers. *Saccharomyces cerevisiae* was cultivated as a facultative anaerobe in 10 ml of YPD medium [1% (w/v) yeast extract, 2% peptone and 2% glucose] containing 20 mM of furfural in a shaking flask at 30°C and 200 r.p.m. When the culture reached the late exponential phase, 1% (v/v) of cell cultures in each tube were transferred to a fresh medium containing 20 mM furfural independently. The cultivation was repeated under the same conditions for up to 50 transfers. The inoculation of 1% (v/v) of the culture into fresh medium in each transfer for repeated batch cultures was considered as 100× dilution of the culture in each transfer. Based on this consideration, the propagation of cells during each culture was formulated as 2^*n*^ = 100, in which *n* was solved for the number of generations in each culture. Therefore, the numbers of generations (*n*) for each transfer and 50 transfers were determined to be ~6.64 and ~332 generations respectively. Three evolved strains were isolated from each of the final cultures by streak‐outs on YPD agar plates. For further experiments, biological duplications of all three evolved strains separately obtained by an independent evolutionary process were utilized.

### Measurement of growth phenotype

To assess growth performance, the parental and evolved strains were cultivated in 100 ml of YPD medium containing various concentrations of furfural ranging from 0 to 40 mM in a shaking flask at 30°C and 200 r.p.m. Cell growth was measured as absorbance at 600 nm (A_600_; Mark Microplate Spectrophotometer; Bio‐Rad, Hercules, CA, USA). For the verification of phenotypic stability, the evolved strains were cultivated in medium without furfural for up to five transfers (~33 generations). The relative growth of the obtained cells was evaluated under furfural exposure.

For the analysis of extracellular metabolites, supernatants obtained after centrifugation at 13 000 r.p.m. for 5 min were filtered through 0.2 μm syringe filters prior to high performance liquid chromatography (HPLC; Agilent 1100; Agilent Technologies, Santa Clara, CA, USA) with a refractive index detector (G1362A; Agilent Technologies). HPLC was carried out on an Aminex HPX‐87H column (H^+^ form; Bio‐Rad) operating at 65°C with 5 mM H_2_SO_4_ as a mobile phase and at a flow rate of 0.5 ml min^−1^ to measure the concentrations of glucose, ethanol, furfural and glycerol. All the analyses were conducted in duplicate. To determine the dry cell mass, 10 ml of culture broth was centrifuged at 13 000 r.p.m. for 5 min at 4°C and washed twice using phosphate‐buffered saline (KH_2_PO_4_ 0.24 g l^−1^, KCl 0.2 g l^−1^, NaCl 8 g l^−1^ and Na_2_HPO_4_ 1.44 g l^−1^ at pH 7.4). The collected cell pellet was then dried using a speed vacuum concentrator (Labconco, Kansas City, MO, USA).

### Sample preparation and intracellular metabolite analysis

Six replicates of the parental and evolved strains (i.e. biological duplications of all three evolved strains separately obtained from independent evolutionary processes) were prepared for metabolite analysis. Culture samples were collected at the early exponential phase, when the effect of furfural still remained. Fast filtration was carried out following the method described in a previous study (Kim *et al*., 2013). In brief, within < 30 s, 1 ml of the collected sample was vacuum‐filtered through a nylon membrane filter (0.45 μm pore size, 30 mm diameter; Whatman, Piscataway, NJ, USA), washed with 5 ml of distilled water at room temperature, rapidly mixed with 20 ml of acetonitrile/water (ACN) mixture (1:1, v/v) at −20°C and frozen in liquid nitrogen. After thawing on ice, the cell‐loaded filters and solvent mixture were vortexed for 3 min for further extraction and centrifuged at 16 100 rcf for 5 min at 4°C. The supernatant (1 ml) was collected and vacuum‐dried using a speed vacuum concentrator. The concentrate was then resuspended in 0.5 ml of ACN mixture to remove the lipids and wax and was dried again.

Prior to GC/TOF MS analysis, the dried metabolite concentrates were treated with a two‐stage derivatization method including methoxyamination with 5 μl of 40 mg ml^−1^ methoxyamine hydrochloride in pyridine (Sigma‐Aldrich) at 30°C for 90 min and silylation with 45 μl of *N*‐methyl‐*N*‐trimethylsilyltrifluoroacetamide (Fluka, Buchs, Switzerland) at 37°C for 30 min. A mixture of fatty acid methyl esters was added to the derivatized metabolites as a retention index marker. GC/TOF MS analysis was performed using an Agilent 7890A GC (Agilent Technologies) coupled with a Pegasus HT TOF MS (LECO, St. Joseph, MI, USA). A 1 μl aliquot of the derivatized metabolite was injected into the GC in splitless mode and was separated on an RTX‐5Sil MS column (30 m × 0.25 mm, 0.25 μm film thickness; Restek, Bellefonte, PA, USA) and an additional 10 m long integrated guard column with temperature programmed at 50°C for 1 min, followed by ramping to 330°C at 20°C min^−1^ and holding for 5 min. The ion source and transfer line temperatures were 250°C and 280°C, respectively, and the ions were generated by a 70 eV electron beam. The mass spectra of the metabolites were acquired in a range of 85−500 *m z* ^−1^ at an acquisition rate of 10 spectra s^−1^.

### Metabolite identification and statistical analysis

The spectra obtained by GC/TOF MS analysis were pre‐processed using ChromaTOF software (ver. 3.34; LECO) and were then reprocessed by BinBase, an in‐house programmed database built for metabolite identification (Skogerson *et al*., 2011). After normalization of each culture by the total peak area, the data sets were analysed by STATISTICA (ver. 7.1; StatSoft, Tulsa, OK, USA) for PCA.

## Conflict of interest

None declared.

## Supporting information


**Fig. S1.** Measurement of growth stability of the evolved *Saccharomyces cerevisiae* by elimination of furfural. Evolved mutant strains were serially sub‐cultured 5 times into a new medium without furfural and the cells were obtained after 24 h of fermentation from each culture. Then, the strains designated as E_1 to E_5 were re‐cultivated in the furfural‐containing medium to compare relative growth. For the evolved strains, the mean values of E_a, E_b and E_c were used.
**Fig. S2.** Cell growth. Both (A) parental and (B) evolved (E_b) *Saccharomyces cerevisiae* were grown in YPD medium containing different amounts of furfural ranging from 0 to 40 mM.
**Fig. S3.** Growth profiles of (A) parental and (B) evolved (E_b) *Saccharomyces cerevisiae* grown in YPD medium containing 20 mM of furfural.
**Table S1.** List of metabolites identified by GC/TOF MS and BinBase analysis of the parental and evolved strains grown in YPD medium with or without 20 mM furfural and classified on the basis of their chemical structures. For the evolved strains, the mean values of E_a, E_b and E_c were used.
**Table S2.** Top 15 metabolites with absolute loading on PC1 and PC2 as determined by PCA. For the evolved strains, the mean values of E_a, E_b and E_c were used.
