# Supplementary material for: Intracellular metabolite profiling of Saccharomyces cerevisiae evolved under furfural
Source: Microb Biotechnol. 2016 Dec 8;10(2):395–404. doi: 10.1111/1751-7915.12465 (PMC5328829; doi:10.1111/1751-7915.12465)
Supplement: Supplementary file 1 — Fig. S1. Measurement of growth stability of the evolved Saccharomyces cerevisiae by elimination of furfural. Evolved mutant strains were serially sub‐cultured 5 times into a new medium without furfural and the cells were obtained after 24 h of fermentation from each culture. Then, the strains designated as E_1 to E_5 were re‐cultivated in the furfural‐containing medium to compare relative growth. For the evolved strains, the mean values of E_a, E_b and E_c were used. Fig. S2. Cell growth. Both (A) parental and (B) evolved (E_b) Saccharomyces cerevisiae were grown in YPD medium containing different amounts of furfural ranging from 0 to 40 mM. Fig. S3. Growth profiles of (A) parental and (B) evolved (E_b) Saccharomyces cerevisiae grown in YPD medium containing 20 mM of furfural. Table S1. List of metabolites identified by GC/TOF MS and BinBase analysis of the parental and evolved strains grown in YPD medium with or without 20 mM furfural and classified on the basis of their chemical structures. For the evolved strains, the mean values of E_a, E_b and E_c were used. Table S2. Top 15 metabolites with absolute loading on PC1 and PC2 as determined by PCA. For the evolved strains, the mean values of E_a, E_b and E_c were used. [file MBT2-10-395-s001.pdf]

Supporting Information

Intracellular metabolite profiling of *Saccharomyces cerevisiae* evolved under furfural

Running title: Metabolomics of evolved *S. cerevisiae*

Young Hoon Jung[1], Sooah Kim[2], Jungwoo Yang[2], Jin-Ho Seo[3], Kyoung Heon Kim[2,*]

[1]School of Food Science and Biotechnology, Kyungpook National University, Daegu 41566, South Korea

[2]Department of Biotechnology, Graduate School, Korea University, Seoul 02841, South Korea

[3]Department of Agricultural Biotechnology and Center for Food and Bioconvergence, Seoul National University, Seoul 08826, South Korea

*Corresponding author: K.H. Kim

Email: khekim@korea.ac.kr; Fax: +82-2-9275-1970; Tel: +82-2-3290-3028

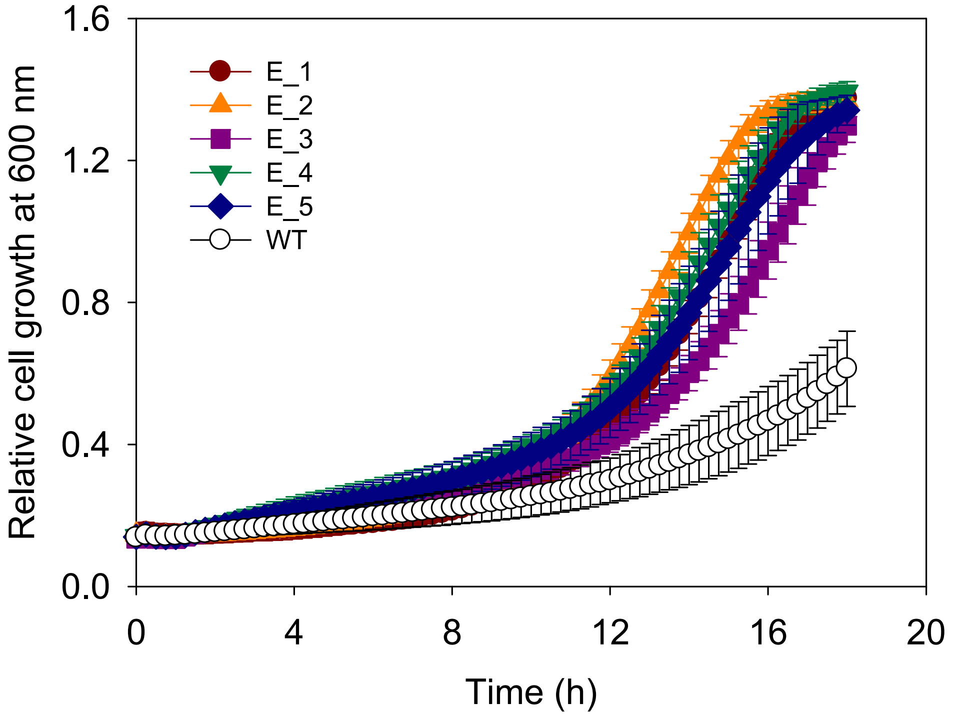

Fig. S1. Measurement of growth stability of the evolved *S. cerevisiae* by elimination of furfural. Evolved mutant strains were serially subcultured 5 times into a new medium without furfural and the cells were obtained after 24 h of fermentation from each culture. Then, the strains designated as E_1 to E_5 were recultivated in the furfural-containing medium to compare relative growth. For the evolved strains, the mean values of E_a, E_b and E_c were used.

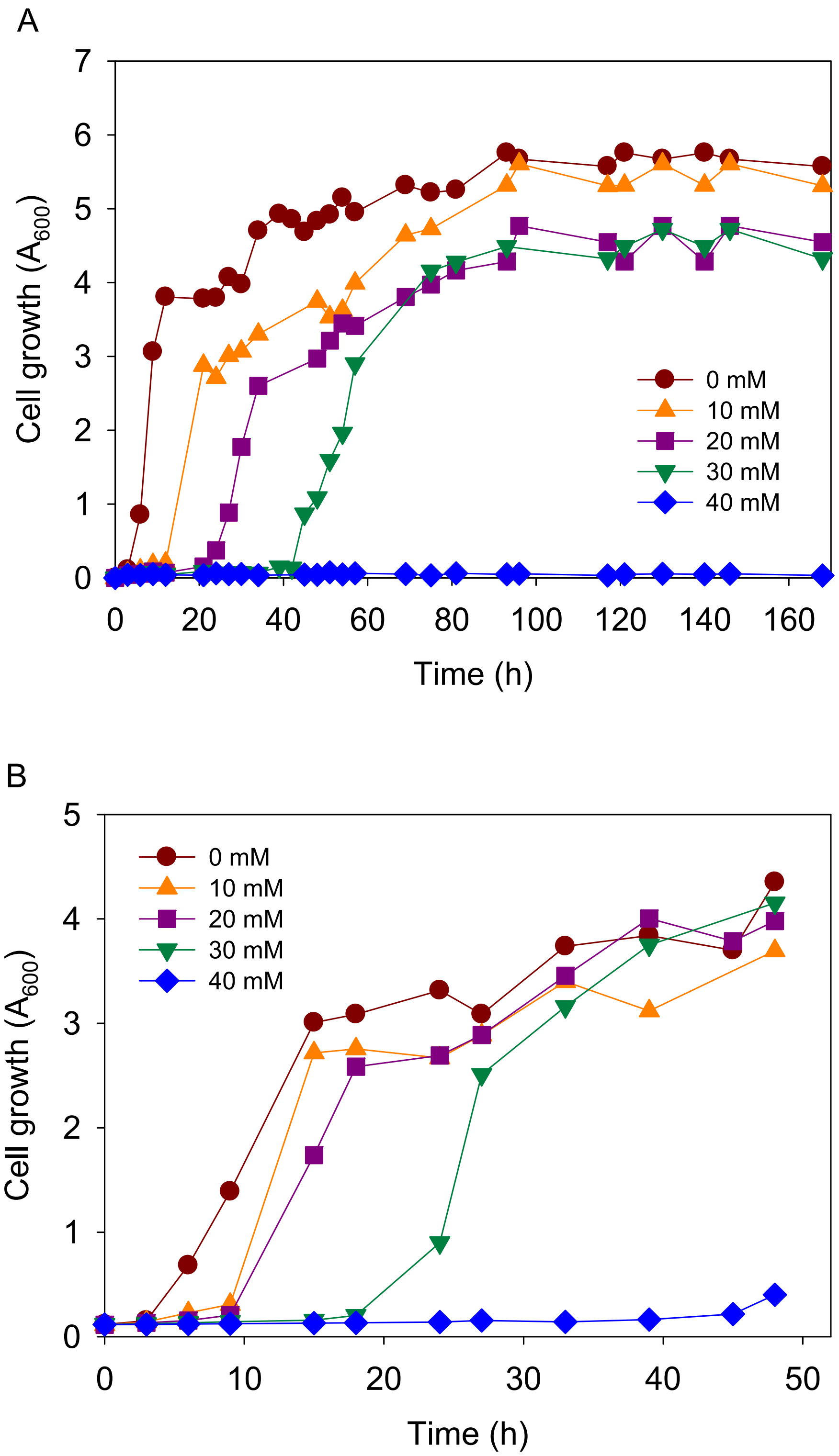

Fig. S2. Cell growth of (A) the parental and (B) evolved (E_b) *S. cerevisiae* grown in YPD medium containing different amounts of furfural ranging from 0 mM to 40 mM.

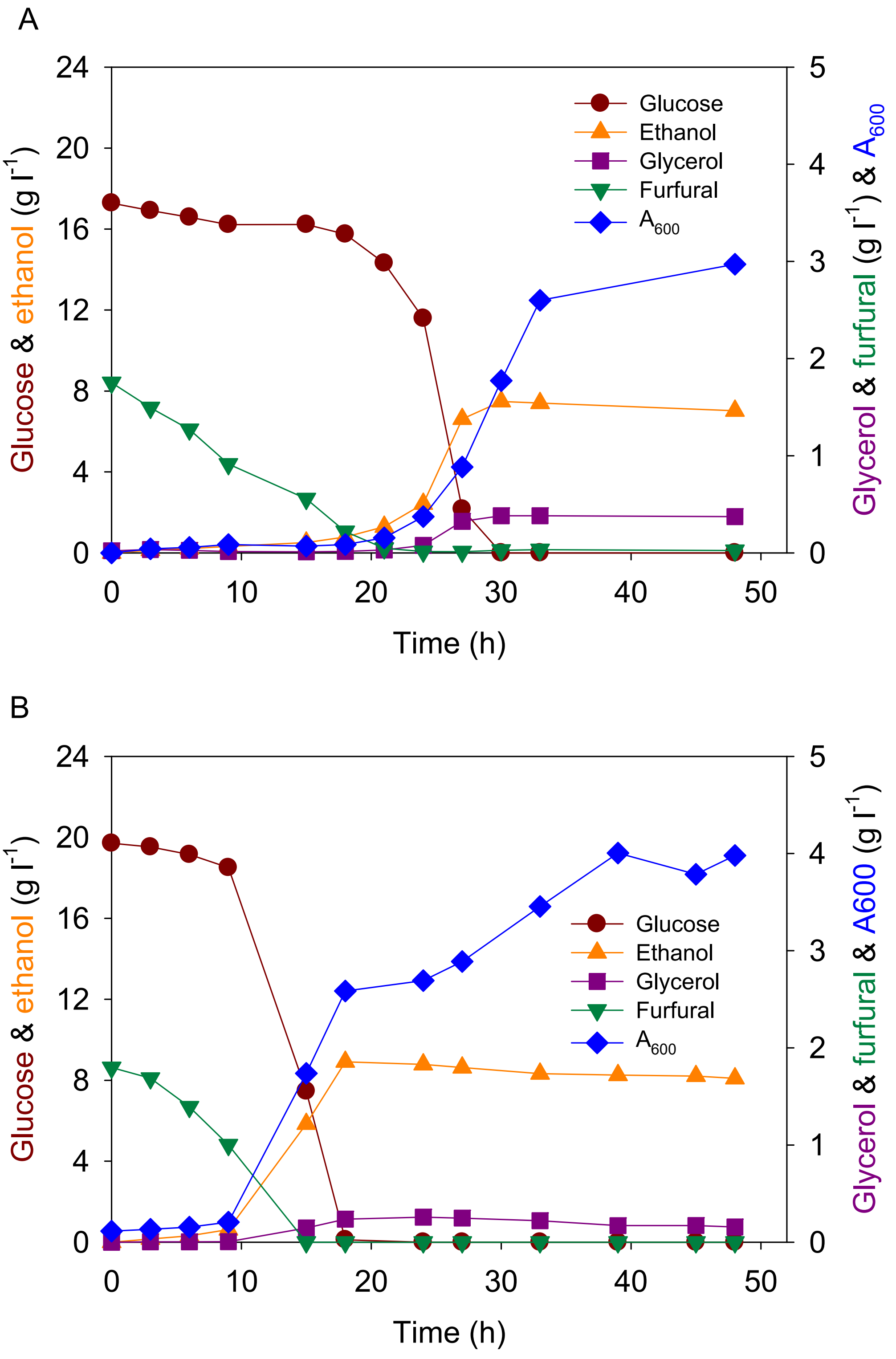

Fig. S3. Growth profiles of (A) the parental and (B) evolved (E_b) *S. cerevisiae* grown in YPD medium containing 20 mM furfural.

Table S1. List of metabolites identified by GC/TOF MS and BinBase analysis of the parental and evolved strains grown in YPD medium with or without 20 mM furfural and classified on the basis of their chemical structures. For the evolved strains, the mean values of E_a, E_b and E_c were used.

**Identified metabolites**	
**Amines and phosphates (22)**	
2-hydroxypyridine	ornithine (ornithine + ornithine 4TMS[b])[a]
3-hydroxypyridine	phosphate
5'-deoxy-5'-methylthioadenosine (MTA)	putrescine
adenosine	saccharopine
adenosine-5'-monophosphate (AMP)	spermidine
carnitine	thymine
cytindine-5'-monophosphate (CMP)	trehalose-6-phosphate
ethanolamine	uracil
guanine	urea
inosine	uridine
L-citrulline	xanthine
**Amino acids (21)**	
alanine	lysine
asparagine (asparagine + asparagine dehydrated)[a]	methionine
aspartate	methionine sulfoxide
cyano-L-alanine	*N*-methylalanine
glutamate	phenylalanine (phenylalanine + phenylalanine minor)[a]
glutamine	proline
glycine (glycine + glycine minor)[a]	serine (serine + serine minor)[a]
isoleucine	threonine (threonine + threonine minor)[a]
L-cysteine	tyrosine

leucine	valine (valine + valine 1TMS[b])[a]
L-homoserine	
**Fatty acids and phenolics (19)**	
1-monopalmitin	octadecanol
2-(4-hydroxyphenyl)ethanol	oleic acid
arachidic acid	palmitic acid
behenic acid	palmitoleic acid
benzoic acid	pelargonic acid
cholic acid	pentadecanoic acid
heptadecanoic acid	salicylaldehyde
lauric acid	squalene
lignoceric acid	stearic acid
myristic acid	
**Organic acids (17)**	
3-hydroxypropionate	lactate
3-phenyllactate	malate
adipate	oxalate
aminomalonate	phenylacetate
beta-hydroxybutyrate	pyrrole-2-carboxylate
citrate	succinate
fumarate	terephthalate
glycerate	xanthurenic acid
glycolate	
**Sugars and sugar alcohols (19)**	
arabitol	mannose
cellobiose	melibiose (melibiose major + melibiose minor)[a]
fructose	myo-inositol
galactose	sucrose
galactinol	tagatose
glucose (glucose 1 + glucose 2)[a]	threitol
glycerol	threose

lactose	trehalose
lactulose	xylose
mannitol	

[a]The total peak intensity of an identified metabolite with more than one peak (peaks were summed to obtain the total)

[b]TMS, the number of hydrogens replaced by trimethylsilylation (TMS) in a partially trimethylsilylated metabolite

Table S2. Top 15 metabolites with absolute loading on PC1 and PC2 as determined by PCA. For the evolved strains, the mean values of E_a, E_b and E_c were used.

**PC1**				**PC2**			
**Metabolite**	**Value (+)**	**Metabolite**	**Value (−)**	**Metabolite**	**Value (+)**	**Metabolite**	**Value (-)**
L-homoserine	0.919	glucose	−0.972	putrescine	0.873	lysine	−0.926
CMP	0.914	galactose	−0.968	arachidic acid	0.716	*N*-methylalanine	−0.878
ornithine	0.893	fructose	−0.898	oxalate	0.695	proline	−0.860
threitol	0.852	adipate	−0.870	xanthurenic acid	0.675	glycine	−0.859
MTA	0.843	tagatose	−0.868	phenylacetate	0.643	aminomalonate	−0.835
cholic acid	0.836	myo-inositol	−0.861	salicylaldehyde	0.641	threonine	−0.803
galactinol	0.815	cyano-L-alanine	−0.844	mannose	0.573	carnitine	−0.769
lactate	0.815	arabitol	−0.821	lauric acid	0.563	succinate	−0.766
palmitoleic acid	0.809	ethanolamine	−0.821	uridine	0.534	valine	−0.706
glutamate	0.809	phenylalanine	−0.809	heptadecanoic acid	0.528	L-citrulline	−0.693
phosphate	0.799	pentadecanoic acid	−0.740	2-(4-hydroxyphenyl) ethanol	0.525	fumarate	−0.691
L-cysteine	0.789	methionine sulfoxide	−0.703	stearic acid	0.523	malate	−0.616

sucrose	0.763	methionine	−0.681	3-hydroxypropionate	0.520	isoleucine	−0.550
oleic acid	0.761	pyrrole-2-carboxylate	−0.652	palmitic acid	0.517	glutamine	−0.531
AMP	0.754	Terephthalate	−0.612	β-hydroxybutyrate	0.499	leucine	−0.530
